# Enhanced Recovery After Surgery Protocol in Emergency Laparotomy: A Randomized Control Study

**DOI:** 10.1055/s-0041-1725156

**Published:** 2021-06-03

**Authors:** Jyoti Sharma, Navin Kumar, Farhanul Huda, Yashwant Singh Payal

**Affiliations:** 1 Department of General Surgery, All India Institute of Medical Sciences, Rishikesh, Uttarakhand, India; 2 Department of Anesthesiology, All India Institute of Medical, Sciences, Rishikesh, Uttarakhand, India

**Keywords:** emergency gastrointestinal surgery, enhanced recovery after surgery protocol, emergency laparotomy

## Abstract

**Introduction** There is established evidence on the role of enhanced recovery after surgery (ERAS) protocols in elective surgeries but its effectiveness in emergency surgeries has been nominally studied. We aimed at studying the feasibility and effectiveness of ERAS protocols in patients undergoing emergency abdominal surgery for intestinal perforation and small bowel obstruction and compare their surgical outcomes with conventional care.

**Materials and methods** This prospective randomized study was performed for a period of 16 months. A total of 100 patients presenting either with intestinal perforation or acute small bowel obstruction were recruited; 50 each in the ERAS and the conventional care groups. The primary outcomes studied were the postoperative length of stay and 30-day morbidity and mortality.

**Results** It was seen that the median (interquartile range) of the duration of hospital stay in the ERAS group was 4 (1) days while it was 7 (3) days in the conventional care group, which was statistically significant (W = 323.000, p ≤ 0.001). Similarly, postoperative morbidities like a chest infection and surgical site infections) were significant in the conventional care group.

**Conclusion** The ERAS protocols are safe and effective in emergency surgeries and result in a better postoperative outcome.

Emergency surgery can be defined as any surgery that deals with an acute threat to life, organ, or tissue due to trauma, any acute disease process, acute exacerbation of the chronic disease, or a complication of a surgical or an interventional procedure. 1 These conditions require early surgical intervention, preferably within 24 hours, as they are associated with a high incidence of morbidity and mortality with rates as high as 80%. 2 These conditions result in an accentuated surgical stress response resulting in an increased catabolic state of the patient and the development of insulin resistance, which is a key element in prolonged recovery and increased morbidity. 3 This theory encouraged the development of a multimodal perioperative care pathway which was designed to reduce this surgical stress response, which would lead to early postoperative recovery as well as reduced length of hospital stay (LOS). This was termed as “enhanced recovery after surgery” (ERAS) or famously known as the “fast track” protocols which were developed by a Danish professor named Henrik Kehlet. 4 5 6 Intestinal obstructions and perforations constitute a major part of acute abdominal conditions presenting to us in our emergency department and are associated with a high incidence of morbidity and mortality if not timely intervened. So, implementation of ERAS protocols in this setting may decrease the enhanced surgical stress response and thus lead to enhanced postoperative recovery. This study was aimed to know the feasibility and efficacy of ERAS protocols in patients undergoing emergency abdominal surgery for intestinal perforation and acute intestinal obstruction.

## Materials and Methods

It was a prospective randomized clinical study performed at All India Institute of Medical Sciences, Rishikesh, India. The duration of this study was 16 months, from January 2019 till April 2020 after ethical clearance from the institutional ethics committee (reference number 282/IEC/PGM/2018), and it was conducted in the Department of Surgery in collaboration with the Department of Anesthesiology of our institution. The trial is registered in Clinical Trials Registry–India (CTRI) with the registration number CTRI/2019/09/021282. The sample size in each group was calculated by PASS 11 (NCSS Statistical Software, Wilton, Connecticut) and length of hospital stay was used for power analysis as it was the only primary outcome that could be predicted. The level of significance was set to 5% and the power of the test as 80%. The ratio of sample size in the control group (conventional care group) and the intervention group (ERAS group) was 1:1 and the total sample size is taken was 100—50 in each group. The inclusion criteria were patients aged18 years and older of either sex presenting with acute intestinal obstruction or intestinal perforation diagnosed preoperatively and planned for emergency laparotomy, patients who gave informed consent for the study, and patients who were hemodynamically stable (i.e., systolic blood pressure equal to or above 90 mm Hg) or falling under American Society of Anesthesiologists (ASA) grade I–IIIe (“e” stands for emergency). The exclusion criteria were patients who were pregnant; patients on chronic steroids; patients with chronic obstructive pulmonary disease; patients with malignant ulcers confirmed by histopathological examination, laparoscopic surgeries, and acute abdominal trauma; and patients who required postoperative intensive care unit (ICU) care. The patients were randomly divided into two groups using computer-generated allocation software (random allocation software) and allocation concealment was done by “sealed envelope technique” to prevent prior knowledge of treatment assignment. The numbers were assigned in strict chronological order and the patients were entered in sequence. The patients in both groups were provided with detailed information about the clinical study and the risks and benefits of both protocols, and a verbal and written informed consent was taken. The patients were then assessed clinically and radiologically where a clinical assessment was done by a detailed history and examination of the patient, routine investigations, preanesthetic checkup, and risk stratification while the radiological assessment was done with an X-ray of the abdomen (erect and supine) and an ultrasonography (USG) of the whole abdomen. Contrast-enhanced computed tomography (CECT)/noncontrast computed tomography (NCCT) of the abdomen was reserved for cases of small and large bowel obstruction where the diagnosis was unclear or to delineate the etiology of obstruction. For the ERAS group, the points that could be applied in emergency laparotomy are included in Table 1 and those for conventional care in Table 2 , respectively. An objective analysis of the concerned parameters was performed with the least risk of bias. Strict adherence to ERAS protocols was followed and compliance of the patients was ensured to both management strategies. All cases underwent exploratory laparotomy through a midline incision under general anesthesia following the standard anesthetic protocol of balanced anesthesia with short-acting anesthetic agents. Peptic ulcer perforations were repaired primarily by modified Graham's omental patch whereas ileal perforation was managed either through primary repair or ileostomy. Intestinal obstructions were treated by band division, adhesiolysis, creating a diversion ileostomy, or colostomy or by resection and anastomosis in case of tubercular strictures. Postoperative management was done according to the defined protocols in each group and the patients were discharged when the discharge criteria (i.e., hemodynamically stable, ambulatory, orally accepting) were met.. The patients were assessed for postoperative complications, LOS, readmission rate, and mortality and were followed till 30 days from surgery. The primary endpoints were the LOS and morbidity and mortality during the first 30 days after surgery which included surgical site infections ([SSIs] both superficial and deep), postoperative ileus, and pulmonary complications including atelectasis and pleural effusion. The secondary endpoints were the time to first flatus, the time elapsed until the resumption of oral feeding, the need for nasogastric tube reinsertion, the need of extra analgesics for pain relief, 30-day readmission, and re-exploration.

**Table 1:** Components of ERAS protocol 5

Preoperative	Intraoperative	Postoperative
Counseling and written informed consent	Balanced anesthesia with short acting drugs	Removal of nasogastric tube in all cases in the immediate postoperative period or within 6 hours postsurgery if the patient's consciousness is decreased
Nil by mouth of 6 hours for solids and 2 hours for clear liquids	Regional anesthesia in the form of epidural analgesia	Removal of urinary catheter on postoperative day 1
Antibiotic prophylaxis	Strict intraoperative fluid management	Early mobilization within 6 hours of surgery
Foley's catheterization and nasogastric tube insertion	Restricted use of intra-abdominal drains	Oral sips after removal of nasogastric tube on postoperative day 0 followed by soft diet on postoperative day 1
Initial resuscitation	Routine use of warmers	Strict fluid management post operatively and early discontinuation of intravenous fluids with resumption of oral feeds
Central venous catheter insertion and IV fluid according to CVP	Postoperative nausea and vomiting prophylaxis	Early removal of abdominal drains on postoperative day 2
		Postoperative nausea and vomiting prophylaxis
		Opioid sparing multimodal analgesia
		Early discharge after the patient is accepting soft diet

Abbreviations: ERAS, enhanced recovery after surgery; IV, intravenous; CVP, Central venous pressure.

**Table 2:** Components of conventional care protocol 6

Preoperative	Intraoperative	Postoperative
Written informed consent	Balanced anesthesia with short acting anesthetic agents	Retaining the nasogastric tube till the patient passes flatus
Nasogastric tube and urinary catheter insertion	No regional anesthesia/analgesia	Allowing oral sips only after passage of flatus and soft diet after passage of feces
Nil by mouth of at least 8 hours	Routine insertion of drains	Nausea and vomiting prophylaxis
Crystalloid infusion and resuscitation	Intraoperative fluid management	Removal of urinary catheters on postoperative day 1
Antibiotic prophylaxis	No routine use of warmers	Perioperative use of opioids
Nausea and vomiting prophylaxis	No routine insertion of central venous catheter	Early mobilization after 12 hours
		discharge once they had passed feces and were taking adequate oral feeds

### Statistical Analysis

The statistical analysis was performed using the IBM SPSS v23 software (Armonk, NY). Discrete variables were represented as counts and percentages. Mean, standard deviation, and median were used for continuous data. Parametric tests (student *t* -test) were used to make group comparisons when the data showed a normal distribution. Nonparametric tests (Wilcoxon test) were used when the data were not normally distributed within the two groups. Fischer's exact test and Pearson's chi-square test were used for categorical data. Odds ratio and relative risk were calculated for dichotomous outcomes. A *p* -value of less than 0.05 was considered statistically significant.

## Results

**Fig. 1:**
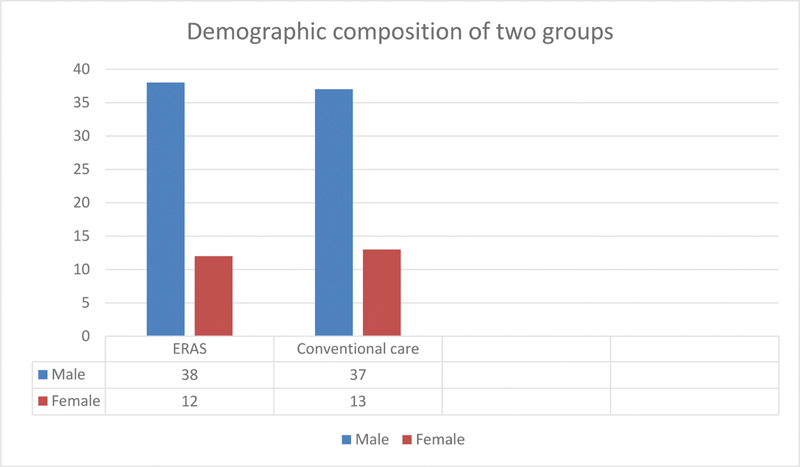
Demographic composition of two groups ( *n* = 100)

A total of 100 consecutive patients presenting with acute intestinal obstruction and intestinal perforation were enrolled in the study who fulfilled the inclusion criteria. Out of the hundred, 75 patients were male and 25 were females ( Fig. 1 ). Both groups were similar in terms of distribution of gender (χ ^2^ = 0.053, *p* = 0.817). These patients were then assigned the respective groups according to the randomization and underwent exploratory laparotomy. Both the ERAS and the conventional groups did not have a normal distribution in terms of age. The age (years) group in ERAS ranged from 18 to 72 years while in the conventional care group it was from 22 to 80 years. There was a significant difference between the two groups in terms of age (W = 952.500, *p* = 0.040), with the median age being highest in the conventional group, that is, 45 years ( Table 3 ). Eighteen out of 37 patients diagnosed with acute intestinal obstruction were managed with ERAS protocols while 19 were managed with conventional care protocol. Thirty-two out of 63 patients diagnosed with intestinal perforation were managed under ERAS protocols while 31 patients were managed with conventional care protocols ( Table 4 ). The surgical outcome of the patients was calculated under the primary and secondary outcomes.

**Table 3:** Comparison of the two groups in terms of age (years) ( *n* = 100)

Age (years)	Group		Wilcoxon test	
	ERAS	Conventional	W	*p* -Value
Mean (SD)	38.10 (15.70)	44.40 (15.97)	952.500	0.040
Median (IQR)	33 (24.75)	45 (25.5)		
Range	18–72	22–80		

Abbreviations: ERAS, enhanced recovery after surgery; IQR, interquartile range; SD, standard deviation.

**Table 4:** Association between group and final diagnosis ( *n* = 100)

Final diagnosis	Group			Fisher's exact test	
	ERAS	Conventional	Total	χ ^2^	*p* -Value
AIO	18 (36.0%)	19 (38.0%)	37 (37.0%)	6.427	0.690
Appendiceal perforation	1 (2.0%)	3 (6.0%)	4 (4.0%)		
Caecal perforation	0 (0.0%)	1 (2.0%)	1 (1.0%)		
Duodenal perforation	2 (4.0%)	2 (4.0%)	4 (4.0%)		
Gastric perforation	3 (6.0%)	3 (6.0%)	6 (6.0%)		
Ileal perforation	5 (10.0%)	5 (10.0%)	10 (10.0%)		
Perforated Meckel's diverticulum	0 (0.0%)	1 (2.0%)	1 (1.0%)		
Prepyloric perforation	21 (42.0%)	14 (28.0%)	35 (35.0%)		
Rectal perforation	0 (0.0%)	2 (4.0%)	2 (2.0%)		
Total	50 (100.0%)	50 (100.0%)	100 (100.0%)		

Abbreviations: AIO, Acute intestinal obstruction; ERAS, enhanced recovery after surgery.

### Analysis of Primary Outcome

The primary outcomes considered in our study were the LOS, perioperative morbidity, and mortality.

1. Duration of hospital stay: A total of 96 patients were evaluated as 4 patients deceased during the postoperative course of hospital stay. The mean (standard deviation [SD]) of the duration of hospital stay in the ERAS group was 5.56 (4.55) days while in the conventional care group it was 8.75 (5.37) days. There was a significant difference between the two groups in terms of duration of hospital stay (W = 323.000, *p* ≤ 0.001), with the median duration being highest in the conventional care group, that is; 7 days ( Table 5 ).
2. Postoperative morbidities: These were paralytic ileus, postoperative nausea and vomiting, pulmonary complications, and SSIs (both superficial and deep).
  - Pulmonary complications/chest infections: There was a significant difference seen between the two groups in terms of occurrence of pulmonary complications (χ ^2^ = 4.828, *p* = 0.028), with the rate being more in the conventional group ( Table 5 ).
  - Paralytic ileus: Ninety-nine patients were evaluated for the occurrence of postoperative ileus which showed a significant difference between both the groups (χ ^2^ = 4.966, *p* = 0.026). A total of 8.0% of the participants in the ERAS group had paralytic ileus as compared with 24.5% of the participants in the conventional care group ( Table 6 ).
  - Postoperative nausea and vomiting (PONV): Multimodal postoperative nausea and vomiting (PONV) prophylaxis was given to patients in the ERAS group and the outcomes were studied; no significant difference was seen between the two groups in terms occurrence of PONV (χ ^2^ = 1.960, *p* = 0.204) ( Table 6 ).
  - Surgical site infections (SSIs): There was a significant difference between the two groups in terms of the distribution of SSIs (χ ^2^ = 5.880, *p* = 0.015), with the conventional group having a larger proportion of SSIs ( Table 6 ).
3. Perioperative mortality: Both the ERAS group and the conventional care group were similar in terms of 30-day mortality risk (χ ^2^ = 0.709, *p* = 0.678) ( Table 6 ).

**Table 5:** Comparison of the two subgroups in terms of primary outcomes of duration of hospital stay and chest infection

		Group		Statistical test	
Duration of hospital stay (days) ( *n* = 96)		**ERAS**	**Conventional**	** W a /χ ^2b^**	***p*** **-Value**
	Mean (SD)	5.56 (4.55)	8.75 (5.37)	323.000 a	< 0.001
	Median (IQR)	4 (1)	7 (3)		
	Range	3–27	4–32		
Chest infection ( *n* = 99)	Present	7 (14.0%)	16 (32.7%)	4.828 b	0.028
	Absent	43 (86.0%)	33 (67.3%)		
	Total	50 (100.0%)	49 (100.0%)		

Abbreviations: ERAS, enhanced recovery after surgery; IQR, interquartile range; SD, standard deviation.

a Wilcoxon test

b Chi-squared test

**Table 6:** Association between two groups in terms of primary outcomes of paralytic ileus, PONV, surgical site infection, and mortality

		Group			Statistical test	
		ERAS	Conventional	Total	χ ^2^	*p* -Value
Paralytic Ileus ( *n* = 99)	Present	4 (8.0%)	12 (24.5%)	16 (16.2%)	4.966 a	0.026
	Absent	46 (92.0%)	37 (75.5%)	83 (83.8%)		
	Total	50 (100.0%)	49 (100.0%)	99 (100.0%)		
PONV ( *n* = 99)	Present	1 (2.0%)	4 (8.2%)	5 (5.1%)	1.960 b	0.204
	Absent	49 (98.0%)	45 (91.8%)	94 (94.9%)		
	Total	50 (100.0%)	49 (100.0%)	99 (100.0%)		
Surgical site infection ( *n* = 98)	Present	18 (36.7%)	30 (61.2%)	48 (49.0%)	5.880 a	0.015
	Absent	31 (63.3%)	19 (38.8%)	50 (51.0%)		
	Total	49 (100.0%)	49 (100.0%)	98 (100.0%)		
Mortality ( *n* = 100)	Present	2 (4.0%)	4 (8.0%)	6 (6.0%)	0.709 b	0.678
	Absent	48 (96.0%)	46 (92.0%)	94 (94.0%)		
	Total	50 (100.0%)	50 (100.0%)	100 (100.0%)		

Abbreviations: ERAS, enhanced recovery after surgery; PONV, postoperative nausea and vomiting

a Chi-squared test

b Fisher's exact test

### Secondary Endpoints

The secondary endpoints that were considered for our study were the time to first flatus, the time elapsed until the resumption of oral feeding, the need for nasogastric tube reinsertion, the need for extra analgesics for pain relief, 30-day readmission, and re-exploration.

1. Time to first flatus: The mean (SD) of day of passing flatus in the ERAS group was 1.78 (0.93) days while it was 2.51 (0.87) days in the conventional care group. The median (interquartile range [IQR]) of day of passing flatus in the ERAS group was similar to that of the conventional group, that is, 2 (1) days ( Table 7 ). There was a significant difference between the two groups in terms of day of passing flatus (W = 633.000, *p* ≤ 0.001), with the mean day of passing flatus being highest in the conventional group, that is; 2.51 days ( Table 7 ).
2. Postoperative pain score and need for extra analgesia: The conventional care group in our study saw a significantly higher pain score and need for extra analgesia as compared with the ERAS group (W = 949.500, *p* = 0.048) ( Table 7 ).
3. Need for nasogastric tube reinsertion: These were similar in both the groups. (χ ^2^ = 0.003, *p* = 1.000) ( Table 8 ).
4. Re-exploration rate: These were similar in both the groups (χ ^2^ = 2.839, *p* = 0.160) ( Table 8 ).
5. Re-admission rate: There was no significant difference between the two groups in terms of 30-day re-admission rates (χ ^2^ = 0.211, *p* = 1.000) ( Table 8 ).

**Table 7:** Comparison of the two groups in terms of secondary outcomes of number of days of passing flatus and pain score ( *n* = 99)

		Group		Wilcoxon test	
		ERAS	Conventional	W	*p* -Value
Day of passing flatus	Mean (SD)	1.78 (0.93)	2.51 (0.87)	633.000	< 0.001
	Median (IQR)	2 (1)	2 (1)		
	Range	1–6	1–5		
Pain score	Mean (SD)	3.48 (1.36)	3.94 (1.28)	949.500	0.048
	Median (IQR)	3 (1)	4 (2)		
	Range	2–7	2–6		

Abbreviations: ERAS, enhanced recovery after surgery; IQR, interquartile range; SD, standard deviation.

**Table 8:** Association between two groups in terms of secondary outcomes of NG reinsertion, reoperation, and readmission

		Group			Fisher's exact test	
		ERAS	Conventional	Total	χ ^2^	*p* -Value
NG reinsertion ( *n* = 98)	Present	3 (6.0%)	3 (6.2%)	6 (6.1%)	0.003	1.000
	Absent	47 (94.0%)	45 (93.8%)	92 (93.9%)		
	Total	50 (100.0%)	48 (100.0%)	98 (100.0%)		
Reoperation ( *n* = 98)	Present	7 (14.0%)	2 (4.2%)	9 (9.2%)	2.839	0.160
	Absent	43 (86.0%)	46 (95.8%)	89 (90.8%)		
	Total	50 (100.0%)	48 (100.0%)	98 (100.0%)		
Readmission ( *n* = 94)	Present	3 (6.4%)	2 (4.3%)	5 (5.3%)	0.211	1.000
	Absent	44 (93.6%)	45 (95.7%)	89 (94.7%)		
	Total	47 (100.0%)	47 (100.0%)	94 (100.0%)		

Abbreviations: ERAS, enhanced recovery after surgery; NG, nasogastric tube.

## Discussion

ERAS protocols have shown promising results in elective surgeries by greatly reducing the postoperative complications and causing early return of bowel function, thus reducing the LOS. But there is meager literature and research in case of emergency surgeries, owing to the assumption that the elements of ERAS cannot be applied to emergency settings. However, recent studies conclude otherwise and most of the elements can be included in emergency surgeries. We reported a reduced LOS along with reduced postoperative complications and faster recovery of bowel function. However, mortality rate, readmission, and re-exploration rates were similar for both groups. On reviewing the initial studies, we found a case-matched study by Lohsiriwat published in 2014 which included 60 patients divided into ERAS and non-ERAS groups in the ratio of 1:2 undergoing emergency resection for obstructive colorectal cancer. 7 He concluded a significantly shorter length of a hospital stay along with no difference in 30-day mortality and readmission rates which were similar to our study. However, he reported a nonsignificant reduction in the incidence of postoperative complications while our study showed a significant reduction in postoperative complications, especially postoperative ileus, SSIs, and pulmonary complications. This was one of the initial studies to establish the effectiveness and feasibility of ERAS protocols in the setting of emergency surgery. A randomized control trial performed by Gonenc et al in 2014 analyzed the feasibility of ERAS protocols in emergency laparoscopic surgery for perforated peptic ulcer and concluded similar results as our study. 8 It also negated the use of nasogastric tube for decompression and delayed oral feeding which was in itself a landmark. Wisely, in 2016, published a retrospective cohort study comparing 370 patients undergoing emergency abdominal surgeries for various diseases before and after the introduction of ERAS protocols and concluded that the ERAS group had significantly fewer patients who required catheters, drains, or postoperative analgesia for more than 2 days. 9 Major postoperative complications like urinary tract infections and chest infections were significantly reduced as was concluded by our study. However, in contrary to our study, Wisely concluded a similar duration of hospital stay in both the ERAS and non-ERAS groups. Another study by Shida et al in 2017 concluded that ERAS protocols resulted in a reduced median hospital stay by 3 days and a comparable rate of readmissions and mortality as concluded by our study, but, in contrast, also reported no significant reduction in postoperative complication rates. 10 In contrast to our study, Tengberg et al, in 2017, published a large single-center study including patients undergoing acute high-risk abdominal surgery and concluded a significant reduction in 30-day mortality rate in the intervention group (15.5% vs. 21.8% in the control group) and a 7.3% reduction in 180-day mortality. 11 Similar conclusion was obtained from the National Emergency Laparotomy Audit (NELA) that has been collecting data on all adult patients undergoing non–trauma-related emergency surgeries in NHS hospitals within England and Wales since 2013. 12 They also reported that since 2013, the national 30-day mortality rate in the United Kingdom has fallen from 11.8 to 9.5%. However, they also showed a reduction in LOS from 19.2 to 15.6 days while our study showed a reduction of hospital stay from 8.75 to 5.56 days. A systematic review published by Paduraru in 2017 showed a significant reduction in LOS by 2 to 3 days in two studies which were consistent with our study along with no effect on the mortality rates. 13 There was no significant increase in readmission rates in any of the studies which was also seen in our study. Shang et al, in 2018, did a multicenter study from China on 839 patients with obstructive colorectal cancer undergoing emergency laparotomy and concluded that the ERAS group had a significantly faster gastrointestinal recovery, fewer complications, and shorter LOS, which was also depicted in our study. 14 Lohsiriwat, in 2019, published another case-matched study where observed the clinical outcome of patients undergoing emergency colectomy and/or proctectomy with ERAS protocol from 2011 to 2017. 15 He observed a reduced LOS which was consistent with his previous study done in 2014 and also with our study. Also, strict adherence to ERAS protocol was associated with lower morbidity. Our study also showed similar results that were consistent with the results produced by a recent meta-analysis by Shahab Hajibandeh, published in January 2020, which compared 6 studies with 1,334 patients. He concluded that ERAS protocols resulted in the earlier return of bowel function and earlier resumption of oral feeds resulting in a shorter LOS. ERAS protocols also resulted in a lower rate of postoperative complications, mainly pulmonary complications, SSIs, and paralytic ileus, which was reciprocated in our study. Similar to our findings, the meta-analysis also concluded that the risk of 30-day mortality, readmissions, and re-exploration was the same in both the groups. This was the first meta-analysis that investigated the literature evaluating the ERAS protocols in emergency settings. 16

Despite the studies depicting the success of ERAS, a recently published Enhanced Perioperative Care for High-Risk Patients (EPOCH) trial failed to demonstrate any of the results which were depicted in our study as well as the studies mentioned earlier. 17 It showed no significant difference in the LOS or readmission rate between the two groups.

## Conclusion

Tailored ERAS protocol is safe and effective in emergency surgery. However, validation of any study requires a repeated measurement of the endpoints which yield consistent values. We would suggest that if a standardized ERAS protocol specific for emergency surgeries could be developed, it would lead to a much better clinical outcome of the patient and also reduce the economic burden of a country due to high hospital costs because of the morbidity associated with these diseases.
